# Inhibition of the deubiquitinase USP5 leads to c-Maf protein degradation and myeloma cell apoptosis

**DOI:** 10.1038/cddis.2017.450

**Published:** 2017-09-21

**Authors:** Siyu Wang, Jiaxiang Juan, Zubin Zhang, Yanyun Du, Yujia Xu, Jiefei Tong, Biyin Cao, Michael F Moran, Yuanying Zeng, Xinliang Mao

**Affiliations:** 1 Jiangsu Key Laboratory for Translational Research and Therapeutics of Neuro-Psycho- Diseases, Department of Pharmacology, College of Pharmaceutical Sciences, Soochow University, Suzhou, Jiangsu 215123, China; 2 Program in Molecular Structure and Function, The Hospital for Sick Children, Department of Molecular Genetics, University of Toronto, Toronto M5G 0A4, Canada; 3 Department of Oncology, Suzhou Municipal Hospital East Campus, Suzhou 215100, China; 4 Jiangsu Key Laboratory of Preventive and Translational Medicine for Geriatric Diseases, Soochow University, Suzhou 215123, China; 5 Key Laboratory of Protein Modification and Degradation, School of Basic Medical Sciences; Affiliated Cancer Hospital & Institute of Guangzhou Medical University, Guangzhou 511436, China

## Abstract

The deubiquitinase USP5 stabilizes c-Maf, a key transcription factor in multiple myeloma (MM), but the mechanisms and significance are unclear. In the present study, USP5 was found to interact with c-Maf and prevented it from degradation by decreasing its polyubiquitination level. Specifically, the 308th and 347th lysine residues in c-Maf were critical for USP5-mediated deubiquitination and stability. There are five key domains in the USP5 protein and subsequent studies revealed that the cryptic ZnF domain and the C-box domain interacted with c-Maf but the UBA1/UBA2 domain partly increased its stability. Notably, MafA and MafB are also members of the c-Maf family, however, USP5 failed to deubiquitinate MafA, suggesting its substrate specificity. In the functional studies, USP5 was found to promoted the transcriptional activity of c-Maf. Consistent with the high level of c-Maf protein in MM cells, USP5 was also highly expressed. When USP5 was knocked down, c-Maf underwent degradation. Interestingly, USP5 silence led to apoptosis of MM cells expressing c-Maf but not MM cells lacking c-Maf, indicating c-Maf is a key factor in USP5-mediated MM cell proliferation and survival. Consistent with this finding, WP1130, an inhibitor of several Dubs including USP5, suppressed the transcriptional activity of c-Maf and induced MM cell apoptosis. When c-Maf was overexpressed, WP1130-induced MM cell apoptosis was abolished. Taken together, these findings suggest that USP5 regulates c-Maf stability and MM cell survival. Targeting the USP5/c-Maf axis could be a potential strategy for MM treatment.

The Maf transcription factors belong to the basic leucine zipper AP-1 family but with distinctive features.^1^ There are seven Maf proteins in human cells including MafA, MafB, c-Maf, MafF, MafG, MafK, and NRL, of which MafA, MafB, and c-Maf are members of the large Maf family because these proteins share a similar structure as a transcription factor specifically including the DNA-binding domain and transcription activation domain.^2^ These transcription factors at the embryonic stage are widely involved in tissue development and cell differentiation, including touch receptor development and macrophage cell differentiation.^2, 3^ In adult, these Maf genes are highly expressed in malignant blood cancers, typically in multiple myeloma (MM) and mantle cell lymphoma.^4^ MM is a class of hematological malignancy derived from plasma cells that secret antibodies. It is reported that >50% of MM cells overexpress c-Maf.^4^ c-Maf leads to myelomagenesis, which is demonstrated in a c-Maf transgenic mice study in which c-Maf transgenic mice develop myeloma-like features at their old age.^5^ In contrast, dominant negative interference with a mutant form of c-Maf markedly decreases the secretion of abnormal immunoglobulin and extends the survival periods of mice bearing MM tumors.^4^ Dexamethasone is a mainstay of anti-MM drug, we previously found that dexamethasone-mediated MM cell apoptosis is associated with c-Maf degradation.^6^ These findings thus suggest c-Maf is a marker of poor prognosis of MM and targeting at c-Maf could be a therapeutic strategy of MM.^7^

Recent investigations demonstrated that c-Maf degradation is processed by the ubiquitin-proteasome pathway,^8^ requiring ubiquitin-activating enzymes, ubiquitin-conjugating enzymes, ubiquitin ligases, and deubiquitinases.^9^ Our recent studies revealed that c-Maf can be ubiquitinated by the ubiquitin-conjugating enzyme UBE2O^10^ and the ubiquitin ligase HERC4.^11^ Both UBE2O and HERC4 are downregulated in MM cells, when they are restored, MM cells expressing c-Maf will undergo apoptosis.^10, 11^ We also found that the ubiquitin-specific peptidase 5 (USP5) antagonizes the biological function of HERC4 in terms of c-Maf polyubiquitination,^11^ but the underlying mechanisms and pathophysiological significance are not clear. In the present study, we found that USP5 stabilizes c-Maf protein by preventing its ubiquitination while inhibition of USP5 leads to c-Maf degradation and MM cell apoptosis.

## Results

### USP5 interacts with c-Maf protein and decreases its polyubiquitination level

**Figure 1:**
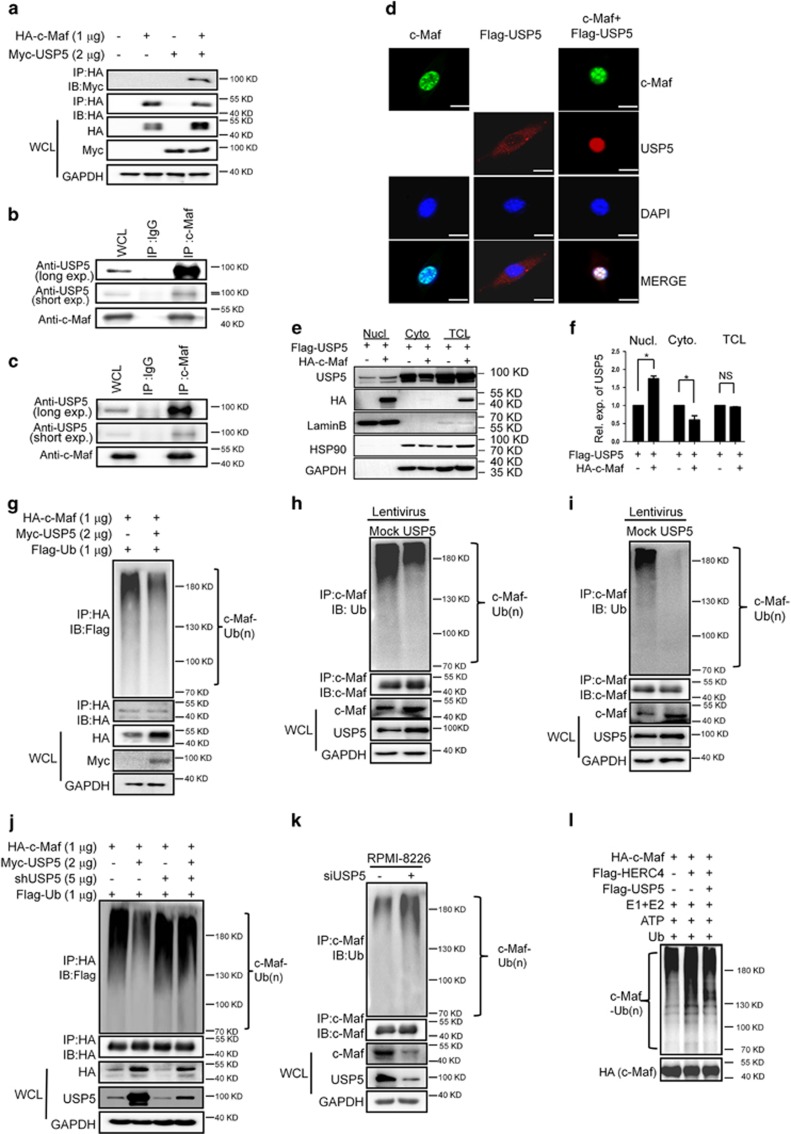
USP5 interacts with c-Maf and decreases its ubiquitination level. (**a**) HEK293T cells were co-transfected with Myc-USP5 and/or HA-c-Maf for 48 h, followed by cell lysate preparation, immunoprecipitation (IP) with an anti-HA antibody and subsequent immunoblotting (IB) with an anti-Myc antibody. (**b**, **c**) Cell lysates from MM cell lines RPMI-8226 (**b**) and LP1 (**c**) were incubated with anti-c-Maf antibody overnight, followed by IB with an anti-USP5 or anti-c-Maf antibody. (**d**) HEK293T cells were transfected with c-Maf, Flag-USP5, or both plasmids. Forty-eight hours later, cells were subjected to the specific antibody staining and confocal analysis. Bar: 50 *μ*M. (**e**) Cells from **d** were subjected to cell lysate preparation and nucleus-cytoplasm cellular fractionation, followed by IB analysis. (**f**) densitometric analysis of USP5 from (**e**) were analyzed by Image J against individual internal controls. (**g**) HEK293T cells were co-transfected with USP5 and c-Maf for 48 h, cell lysates were prepared for IP with an anti-HA antibody and IB assay with indicated antibodies. (**h**–**i**) MM cell lines RPMI-8226 (**h**) and LP1 (**i**) were infected with lentiviral USP5 or mock for 96 h, followed by cell lysate preparation and IP/IB assays with specific antibodies as indicated. (**j**) HEK293KT cells were co-transfected with c-Maf, Ub, and USP5 plasmids with or without shUSP5, 72 h later, cell lysates were prepared for the IP/IB assays. (**k**) RPMI-8226 cells were transfected with siUSP5 for 72 h, followed by IP and IB as indicated. (**l**), IP-purified HA-c-Maf, and Flag-HERC4 were incubated with E1, E2, ATP, and Ub with or without USP5. When the reaction was stopped, the reactions were subjected to IB against c-Maf

Our previous studies showed that USP5 was present in the c-Maf interactome and prevented c-Maf polyubiquitination.^11^ To confirm this finding, USP5 and c-Maf were co-transfected into HEK293T cells for 48 h before being lyzed for immunoblotting (IB) assay. As shown in Figure 1a, USP5 was found in the immunoprecipitates of c-Maf. This interaction was also found in both RPMI-8226 and LP1 MM cells (Figures 1b and c). To view this physical interaction, c-Maf and USP5 were co-transfected into HEK293T cells for 48 h, followed by immunofluoresence analysis. As shown in Figure 1d, c-Maf was found in the nuclei as expected, and USP5 was mainly found in cytosol. Notably, USP5 was mainly found in the nuclei of cells co-transfected with c-Maf (Figures 1e and f). Therefore, USP5 interacted with c-Maf and its cellular distribution was affected by c-Maf.

Because USP5 is a putative enzyme to prevent protein ubiquitination,^12^ we wondered whether USP5 deubiquitinated c-Maf. To this end, USP5 and c-Maf plasmids were co-transfected into HEK293T cells, followed by immunoprecipitation/immunoblotting (IP/IB) assay. As shown in Figure 1g, addition of USP5 decreased the polyubiquitination levels of c-Maf. This finding was confirmed on endogenous c-Maf in MM cell lines RPMI-8226 and LP1 (Figures 1h and i). To convince these findings, USP5 was knocked down by its specific short hair interfering RNA (shRNA) in both HEK293T cells and RPMI-8226 cells. As shown in Figure 1j, USP5 decreased c-Maf ubiquitination in HEK293T cells, but when shUSP5 was introduced, USP5-induced deubiquitination of c-Maf was abolished. In the MM cell line, USP5 knockdown increased c-Maf ubiquitination (Figure 1k). Finally, c-Maf deubiquitination by USP5 was verified in the in-tube ubiquitination assay. As shown in Figure 1l, when USP5 was added to the purified c-Maf proteins mixed with E1/E2, Ub, ATP, and HERC4, an E3 ligase of c-Maf,^11^ c-Maf ubiquitination level was markedly decreased. All the above results thus concluded that USP5 interacted with c-Maf and decreased its polyubiquitination level.

### USP5 does not prevent the polyubiquitination of MafA

**Figure 2:**
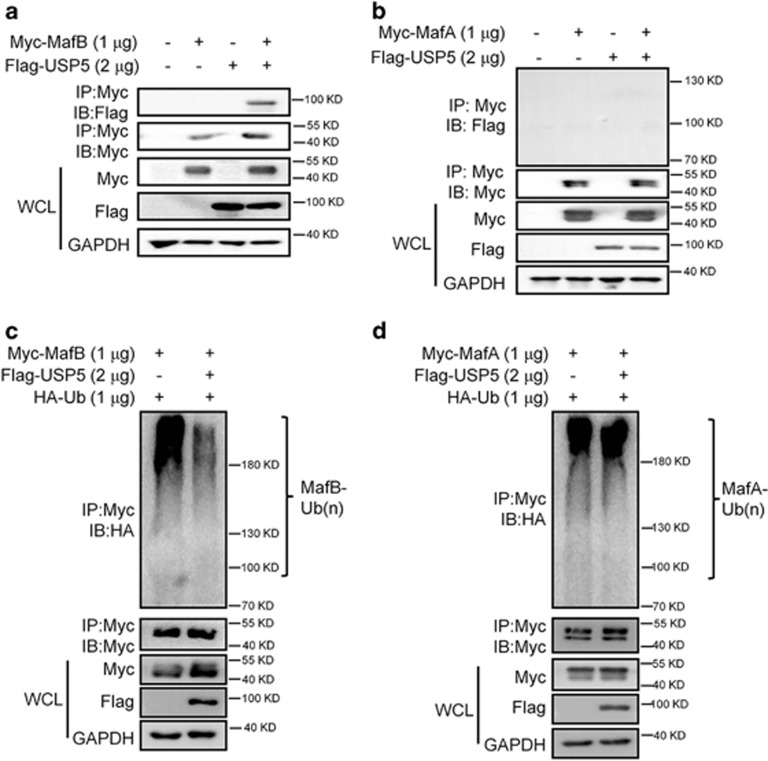
USP5 decreases the polyubiquitination levels of MafB but not MafA. (**a, b**) HEK293T cells were co-transfected with USP5 and MafB (**a**) or MafA (**b**), 48 h later, cell lysates were prepared for IP with a specific antibody and IB assays with indicated antibodies. (**c, d**) HEK293T cells were co-transfected with USP5 and MafB (**c**) or MafA (**d**) for 48 h, cell lysates were then prepared for the IP/IB assays with indicated antibodies

Because MafA, MafB, and c-Maf belong to the large Maf family and they share a high similarity in their amino-acid sequences and biological functions,^1^ we wondered whether USP5 could interact with MafA and MafB. To this end, a USP5 plasmid was co-transfected into HEK293T cells with MafA and MafB plasmids, respectively. The whole-cell lysates were then prepared for the IP/IB assays. As shown in Figures 2a and b, USP5 was found in the MafB but not in the MafA- precipitates. Next, we evaluated the ubiquitination levels of these two proteins. As shown in Figures 2c and d, USP5 led to decreased polyubiquitination on MafB but not on MafA when USP5 was co-transfected with MafA and MafB. These findings were consistent with the interaction analysis (Figures 2a and b). Therefore, USP5 selectively interacted with c-Maf and MafB and modulated their ubiquitination modification but had no effects on MafA ubiquitination.

### USP5 increases Maf protein stability

**Figure 3:**
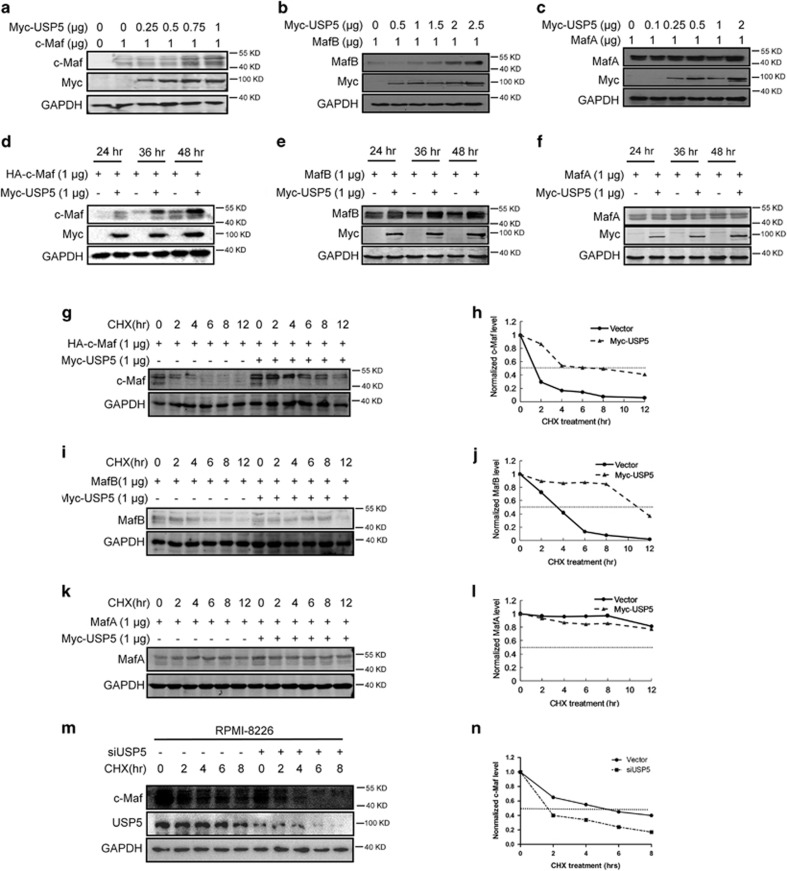
USP5 increases the stability of c-Maf and MafB but not MafA. (**a–c**) HEK293T cells were transfected with Myc-USP5, HA-c-Maf (**a**), MafB (**b**), or MafA (**c**) for 48 h. Cell lysates were subjected to IB with indicated antibodies. (**d, e**) HEK293T cells were transfected with c-Maf (**d**), MafB (**e**), or MafA (**f**) along with increased USP5 for indicated time periods. Cell lysates were used for immunoblotting to determine the Maf protein levels. (**g**–**l**) HEK293T cells were transfected with MafA, MafB, or c-Maf with or USP5 for 24 h, followed by CHX treatment for 0–12 h. Cell lysates were used for immunoblotting to measure the protein levels of c-Maf (**g**), MafB (**i**), or MafA (**k**). The intensities of c-Maf, MafB, and MafA protein bands compared to GAPDH were shown in **h**, **j** and **l**, respectively. (**m**–**n**) MM cell line RPMI-8226 were infected with shUSP5 for 72 h, followed by CHX treatment for 0–8 h. Cells were then subjected to the IB analyses against specific antibodies (**m**) and the intensities of c-Maf against GAPDH was shown in **n**

The above studies showed that USP5 interacted with c-Maf and MafB and reduced their polyubiquitination levels. Because polyubiquitination is a key factor in protein degradation, we wondered the specific effects of USP5 on Maf protein stability. To this end, USP5 was co-transfected with Maf plasmids followed by IB assays. The results demonstrated that USP5 increased the protein levels of c-Maf (Figures 3a and d) and MafB (Figures 3b and e) in a time- and concentration-dependent manner, but USP5 failed to modulate MafA protein stability neither in the increased concentrations of USP5 (Figure 3c) or in the extended co-transfection duration (Figure 3f). To confirm this finding, USP5 was co-transfected with c-Maf, MafA, or MafB, followed by the treatment of CHX, an inhibitor of protein synthesis *de novo*. The IB assay showed that USP5 extended the half-life of c-Maf (Figures 3g and h) and MafB (Figures 3i and j) but not MafA (Figures 3k and l). To confirm this finding, USP5 was also knocked down in RPMI-8226, a typical MM cell line, followed by CHX treatment and c-Maf measurement. The result showed that knockdown of USP5 accelerated c-Maf degradation (Figures 3m and n). Taken all the above results together, we concluded that USP5 increased the stability of c-Maf and MafB by reducing their polyubiquitination levels.

### The 308th and the 347th lysine residues are target sites of USP5 in preventing c-Maf from polyubiquitination

**Figure 4:**
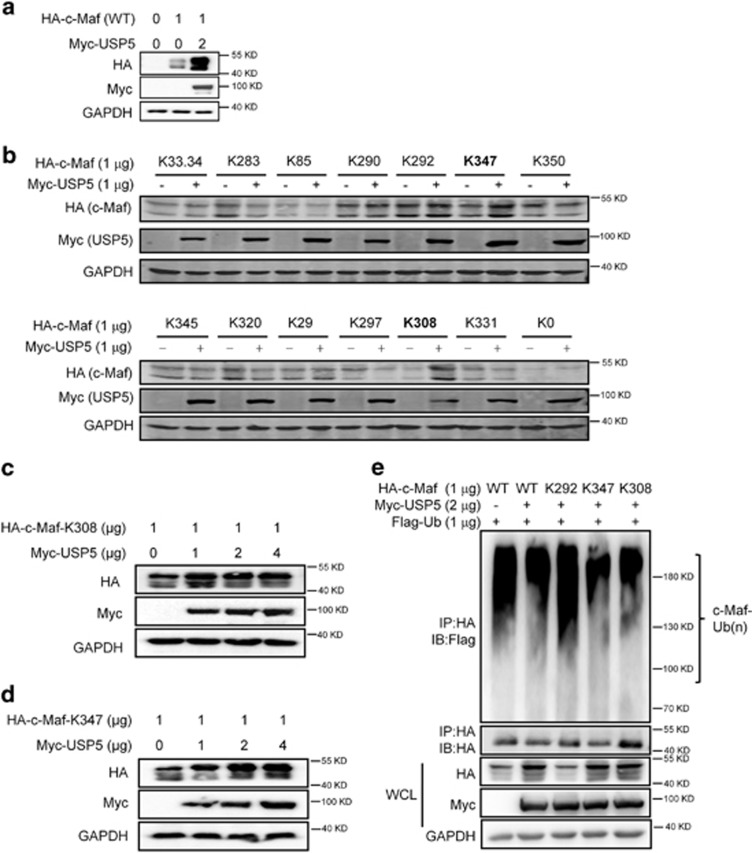
K308 and K347 at c-Maf are the target sites of USP5 in preventing c-Maf ubiquitination. (**a**) HEK293T cells were transfected with a wild-type (WT) plasmid for 48 h, followed by cell lysate preparation and IB with individual antibodies. (**b**) A Myc-USP5 plasmid was co-transfected into HEK293T cells with specific HA-c-Maf mutant plasmids for 48 h, followed by immunoblotting. (**c**, **d**) HEK293T cells were transfected with HA-c-Maf-K308 (**c**) or HA-c-Maf-K347 (**d**) along with increased USP5. Forty-eight hours later, cells were harvested for IB to determine the c-Maf protein levels. (**e**) HEK293T cells were transfected with HA-tagged wild-type (WT) or mutant c-Maf, followed by the IP/IB assays with indicated antibodies

The lysine residues are ubiquitin acceptor sites, to find out which lysine residue was critical for USP5-targeted c-Maf protein deubiquitination, a series of c-Maf mutants contained one single intact lysine residue were generated by replacing all other lysine (K) to arginine (R)^8^ and these c-Maf mutants were then co-transfected with USP5 to evaluate the stability of each mutant c-Maf using IB assays. There was no doubt that USP5 increased c-Maf protein as shown in Figure 4a. In the assays for c-Maf mutants with a single intact lysine residue, only the K308 and K347 mutants (in which c-Maf maintained only the 308th or the 347th lysine residues) could be increased by USP5 (Figure 4b). Moreover, USP5 upregulated the protein levels of the K308 and K347 c-Maf mutants in a concentration-dependent manner (Figures 4c and d). These results suggested that the 308th and 347th lysine residues could be the main target sites of USP5 in deubiquitinating c-Maf. To confirm this hypothesis, the wild-type (wt), K308, or K347 plasmids were co-transfected into HEK293T cells, followed by IP/IB assays. The K292 c-Maf mutant that was not affected by USP5 (Figure 4b) was used as a negative control. As expected, the protein levels of wt, K308, and K347 c-Maf were increased by USP5, which was consistent with the finding on c-Maf ubiquitination level (Figure 4e). USP5 failed to decrease the ubiquitination level on K292 c-Maf, but the ubiquitination levels on wt, K308 and K347 c-Maf were decreased (Figure 4e). Therefore, the ubiquitination at the 308th and the 347th lysine residues could be the major acting sites of USP5 to prevent c-Maf ubiquitination.

### The UBA1/UBA2 domain in USP5 partly prevents Maf protein degradation

**Figure 5:**
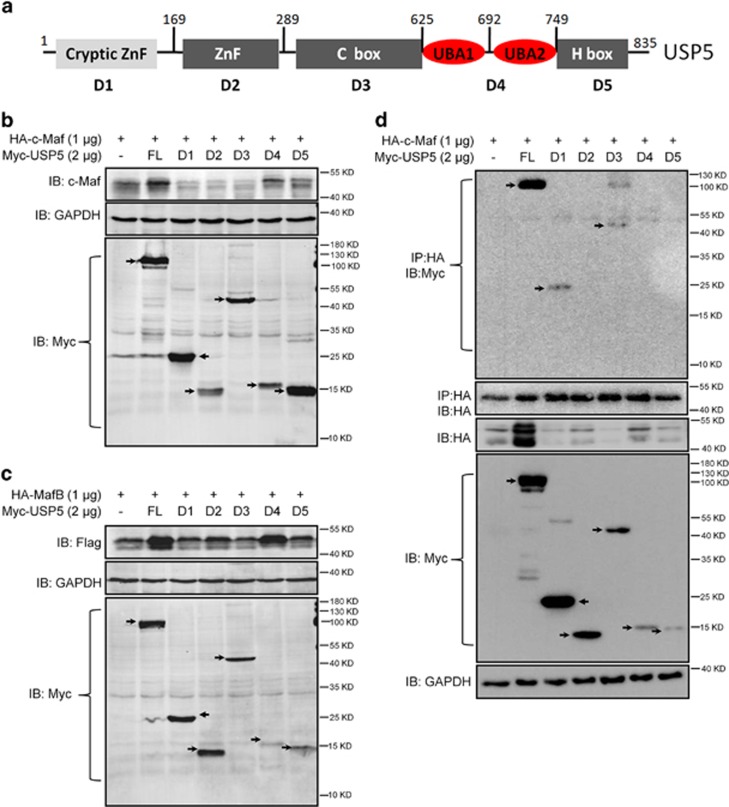
The UBA1–UBA2 domain partly maintains c-Maf stability. (**a**) The scheme of USP5 protein structure and domains. (**b**, **c**) HEK29T cells were transfected with HA-c-Maf (**b**) or MafB (**c**) along with individual Myc-USP5 domains. Forty-eight hours later, cell lysates were prepared for IB with specific antibodies as indicated. Arrows indicate individual domain proteins. FL: full-length. (**d**) c-Maf and Myc-USP5 domains were co-transfected into HEK293T cells for 48 h, followed by cell lysate preparation, IP and IB with specific antibodies as indicated. Arrows indicate individual domain proteins

USP5 is a large protein composed of five specific domains, including the cryptic ZnF domain (D1, aa. 1–168), the ZnF domain (D2, aa. 169–289), the C-Box domain (D3, aa. 290–624), the UBA1/UBA2 domain (D4, aa. 625–749) and the H box domain (D5, aa. 750–835) as shown in Figure 5a. To find out which domain was critical for the action of USP5 on Maf protein stability, constructs of these domains were co-transfected with c-Maf or MafB, followed by determination of Maf protein stability. The result showed that the full-length USP5 increased c-Maf (Figure 5b) and MafB (Figure 5c) proteins. Compared with the D1, D2, D3, and D5 domains that failed to increase Maf proteins, the UBA1/UBA2 domain (D4) partly increased the protein levels of both c-Maf (Figure 5b) and MafB (Figure 5c). This result was probably associated with the biological function of the UBA domain, which is believed to interact with ubiquitin molecules,^13^ thus preventing Maf protein ubiquitination. However, the IP assay revealed that it was the cryptic ZnF domain and the C-Box domain but not the D4 (UBA1/UBA2) domain that interacted with c-Maf (Figure 5d). The discrepancy between D4 and c-Maf interaction (Figure 5d) and c-Maf stability (Figure 5b) was probably because the interaction between c-Maf and D4 was too quick to be captured. These results suggested that specific domains in the USP5 were required for its interaction and protein stabilization.

### USP5 is overexpressed in MM cells

**Figure 6:**
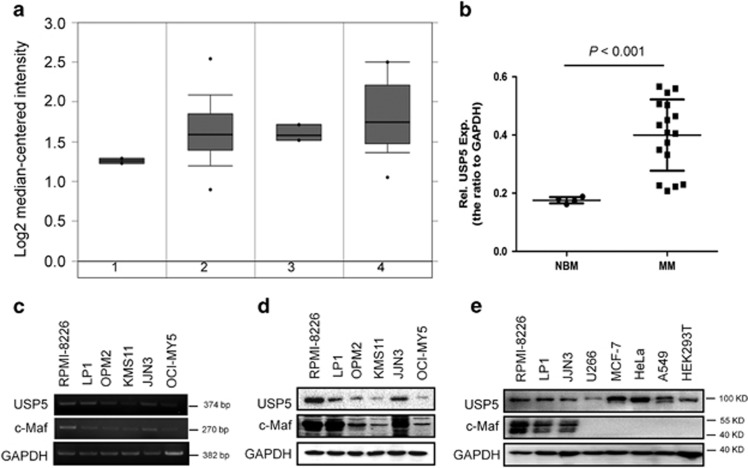
USP5 is highly expressed in MM cells in association with c-Maf. (**a**) Expression of USP5 in primary MM bone marrow cells analyzed by DNA microarray. (1) Monoclonal gammopathy of undetermined significance; (2) Multiple myeloma; (3) Plasma cell leukemia; (4) Smoldering myeloma. (**b**) USP5 mRNA expression levels in normal bone marrow cells (NBM) and myeloma cells (MM). (**c**) the mRNA levels of USP5 and c-Maf in various MM cell lines assayed by RT-PCR. (**d**) USP5 and c-Maf protein expressions in various MM cell lines were evaluated by IB. (**e**) Cell lines with various origins were subjected to USP5 and c-Maf analysis

c-Maf is the major member in the Maf family and it was found in >50% of MM cell lines and primary MM cells.^4^ To find out the expression profile of USP5 in MM cells, we performed an online database analysis. As shown in Figure 6a, the mRNA levels of USP5 was markedly increased in malignant plasma cells including MM, plasma cell leukemia (PCL) and smoldering myelomas (SMM), in contrast, its expression was relatively low in patients of monoclonal gammopathy of undetermined significance, the early stage of MM. To confirm this finding, primary bone marrow cells from healthy adult donors and MM patients were subjected to RT-PCR and densitometry analysis. As shown in Figure 6b, the USP5 levels in MM patients were significantly higher than those in healthy adults. These results suggested that USP5 was induced in MM cells. To solidify this finding, a panel of MM cell lines were analyzed. As shown in Figure 6c, USP5 was highly expressed in some cell lines, including JJN3, LP1, and RPMI-8226. Interestingly, c-Maf protein was high in these cell lines (Figure 6d) in accordance with the expression of USP5. To view whether c-Maf was also expressed in other cell lines with various origins, lung cancer (A549), breast cancer (MCF-7), cervical cancer (HeLa), and HEK293T cells were evaluated. As shown in Figure 6e, USP5 remained a high level in almost all cell lines examined including HEK293T, but c-Maf was only found in MM cell lines. Notably, although c-Maf mRNA was found in most of MM cell lines, its protein levels were consistent with USP5 expression, which suggested that there was an association between c-Maf protein levels and USP5 expression in MM cells.

### USP5 modulates c-Maf transcriptional activity

**Figure 7:**
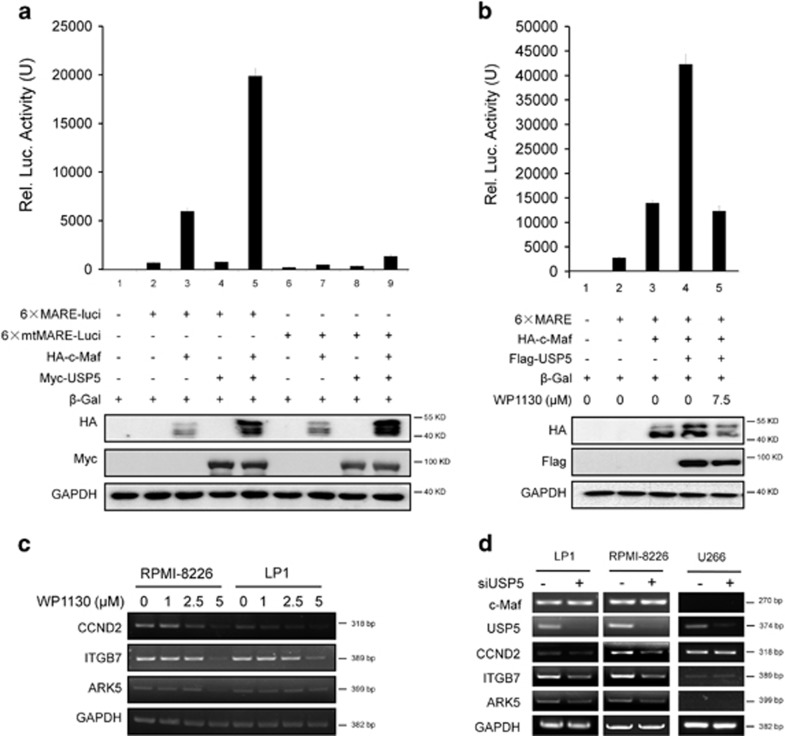
USP5 modulates c-Maf transcriptional activity. (**a**) The MARE.Luci or mtMARE.Luci plasmids were co-transfected with c-Maf or USP5 for 48 h followed by luciferase activity measurements and IB assays. (**b**) Cells transfected with c-Maf and USP5 were treated with WP1130 for 12 h, followed by luciferase activity measurement and IB assays. (**c**) RPMI-8226 and LP1 cells were treated with WP1130 for 12 h, followed by total RNA extraction and RT-PCR analysis. (**d**) LP1, RPMI-8226, and U266 cells were transfected with siUSP5 for 48 h, followed by RT-PCR for indicated genes

c-Maf is a basic zipper transcription factor and modulates the transcription of several key genes in MM. The above studies had demonstrated that USP5 stabilized c-Maf, we therefore wondered whether this action modulated c-Maf biological function. To this end, a firefly luciferase reporter was constructed driven by the 6 × MARE (Maf Recognition Element) or the 6 × mutant MARE (mtMARE).^4^ These reporter plasmids were transfected into HEK293T cells along with c-Maf, USP5, or both, followed by luciferase activity measurement. As shown in Figure 7a, the activity of mtMARE-driving luciferase was very low and it was not markedly affected by c-Maf or USP5. However, c-Maf markedly upregulated the activity of MARE.Luci, and USP5 significantly boosted the effects of c-Maf on MARE.Luci although USP5 alone failed to modulate MARE.Luci. Therefore, these results suggested that c-Maf was the target of USP5.

WP1130 was reported to act as an inhibitor of USP5 and other deubiquitinases,^14^ to further evaluate the regulatory activity of USP5 on c-Maf, WP1130 was added to c-Maf- and USP5-expressing cells transfected with MARE.Luci, followed by luciferase activity measurement. As shown in Figure 7b, WP1130 significantly decreased c-Maf protein and suppressed the transcriptional activity in terms of luciferase reporter expression under the control of MARE. Therefore, these assays demonstrated that WP1130 suppressed c-Maf transcriptional activity. To confirm this action of WP1130, MM cell lines RPMI-8226 and LP1 were incubated with WP1130 for 12 h, followed by RT-PCR assay. As showed in Figure 7c, WP1130 suppressed the transcription of CCND2, ITGB7 and ARK5, downstream genes under c-Maf control,^2, 4^ particularly at a concentration of 5 *μ*M. Consistent with this finding, when USP5 was knocked down in these MM cells, the specific RNA of all three genes were suppressed. In contrast, the transcription levels of these genes were not affected by siUSP5 in U266 cells that lack c-Maf (Figure 7d). To be noted, siUSP5 had no effects on c-Maf at the RNA level (Figure 7d). All these results thus indicated that USP5 modulated c-Maf transcriptional activity by affecting its ubiquitination and protein stability.

### Inhibition of c-Maf or USP5 leads to myeloma cell apoptosis

**Figure 8:**
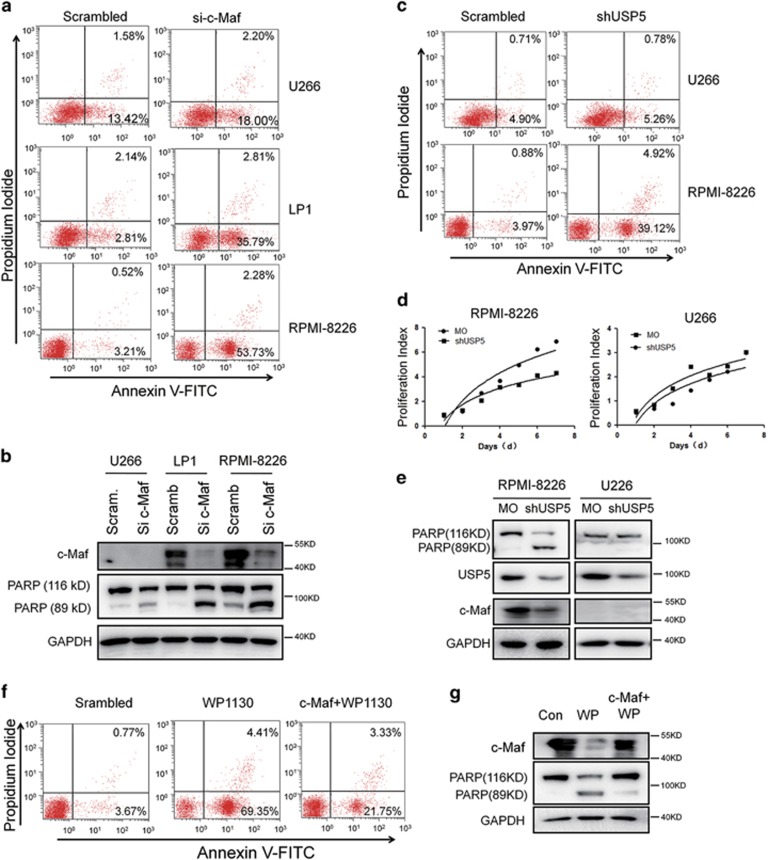
Knockdown of USP5 or c-Maf leads to apoptosis of MM cells expressing c-Maf. (**a**, **b**) RPMI-8226, LP1 or U266 cells were transfected with c-Maf siRNA or scrambled (MO) for 48 h followed by Annexin V/PI staining and FACS analysis (**a**) and IB assays for indicated antibodies (**b**). (**c**–**e**) U266 and RPMI-8226 cells were infected with shUSP5 for 72 h, followed by Annexin V/PI staining and FACS analysis (**c**), MTT assay (**d**), or IB against indicated antibodies (**e**). (**f, g**) RPMI-8226 cells were transfected with a c-Maf plasmid for 48 h followed by WP1130 treatment for 12 h. Cells were then subjected to Annexin V/PI staining and FACS (**f**) and IB assays (**g**), respectively

c-Maf is an oncogenic transcription factor,^7^ and previous studies have demonstrated that c-Maf promotes myeloma formation,^5^ in contrast, interference with c-Maf delays MM growth.^4^ However, whether inhibition of c-Maf induces MM cell apoptosis was not known. Therefore, we first evaluated the effects of MM cell apoptosis after c-Maf was silenced. RPMI-8226, LP1, and U266 cells were infected with c-Maf siRNA (si-c-Maf), followed by Annexin V staining and IB assay. As shown in Figure 8a, si-c-Maf led to apoptosis of both RPMI-8226 and LP1 cells that express c-Maf, however, U266 failed to undergo apoptosis in the presence of si-c-Maf because it lacks c-Maf. Consistent with this finding, IB assay showed c-Maf siRNA resulted in c-Maf knockdown and PARP cleavage, a hallmark of apoptosis (Figure 8b). Because USP5 stabilized c-Maf, we next wondered whether inhibition of USP5 led to MM cell apoptosis via c-Maf. To this end, MM cell lines RPMI-8226 and U266 were infected with lentiviral shUSP5, followed by Annexin V staining/flow cytometry and IB assay. As shown in Figure 8c, shUSP5 led to apoptosis of RPMI-8226 but not U266. Consistent with this result, shUSP5 inhibited the proliferation of RPMI-8226 but not U266 (Figure 8d). This difference between RPMI-8226 and U266 was evaluated by IB assay. As shown in Figure 8e, RPMI-8226 but not U266 cells expressed c-Maf, c-Maf was thus a key player in shUSP5-induced MM cell apoptosis. To confirm this hypothesis, RPMI-8226 was transfected with a c-Maf plasmid, followed by WP1130 treatment. As shown in Figures 8f and g, c-Maf overexpression markedly prevented MM cell apoptosis induced by WP1130. Therefore, c-Maf was a key factor in USP5-induced MM cell survival.

### Targeting at USP5 is a therapeutic strategy for MM

**Figure 9:**
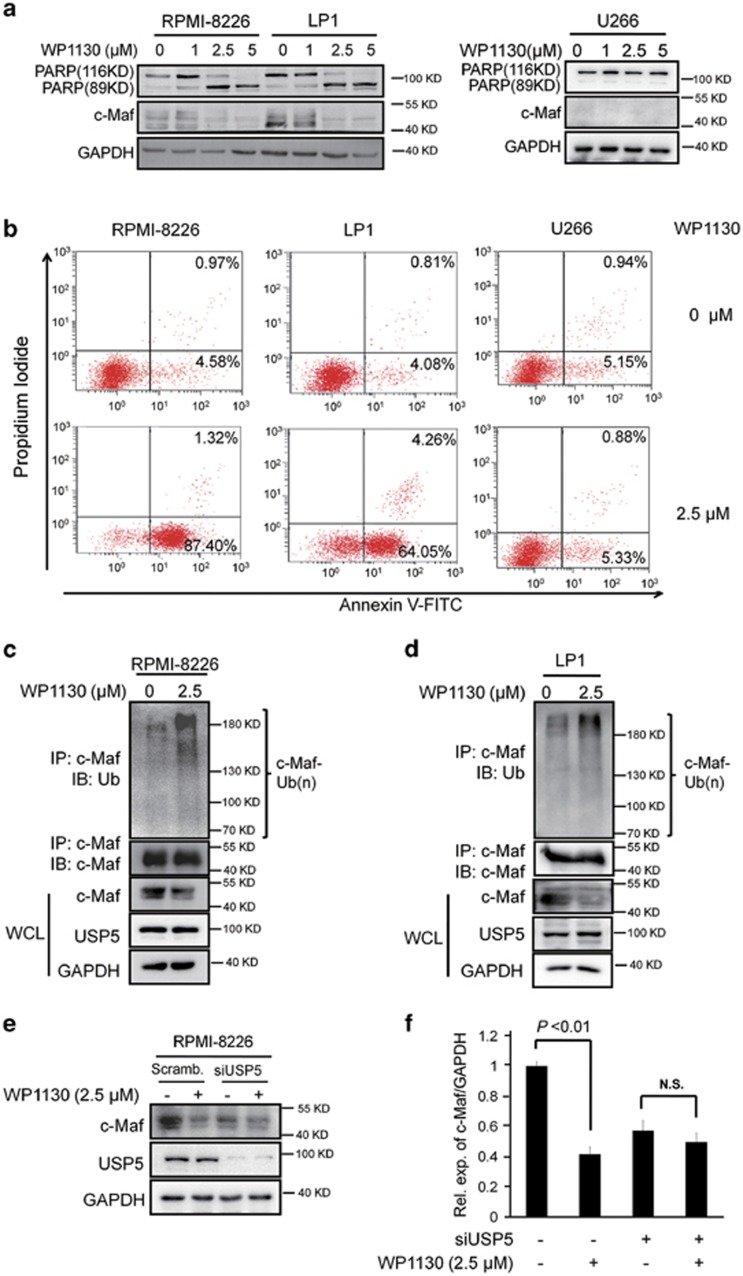
Suppression of the USP5/c-Maf axis leads to MM cell apoptosis. (**a, b**) MM cell lines RPMI-8226, LP1 and U266 were treated with WP1130 at indicated concentrations for 12 h, followed by IB assay (**a**) or Annexin V/PI staining and FACS assay (**b**). (**c, d**) RPMI-8226 (**c**) and LP1 (**d**) cells were treated with WP1130 for 12 h, followed by IP/IB assay. (**e**–**f**) RPMI-8226 cells were transfected with siUSP5 or scrambled siRNA for 72 h, followed by WP1130 treatment for 12 h. The cell lysates were subjected to IB assay (**e**). The ratios of c-Maf over GAPDH from (**e**) were reported. N.S., not significant

The above studies have generated solid data to support the hypothesis that there was a USP5/c-Maf axis in MM that promotes MM cell proliferation and survival. Targeting at USP5/c-Maf could be an effective treatment to induce MM cell death. To test this hypothesis, WP1130, an inhibitor of Dubs including USP5,^14^ was utilized as the tool agent for this study. As shown in Figure 9a, WP1130 treatment led to c-Maf degradation and PARP cleavage in both RPMI-8226 and LP1 cells that express c-Maf but not in U266 that lack c-Maf. To confirm this finding, all three cell lines treated with WP1130 were subjected to Annexin V staining and flow cytometric analysis. As shown in Figure 9b, WP1130 markedly increased the Annexin V-positive fractions in RPMI-8226 and LP1, but not in U266 cells. Because c-Maf ubiquitination and degradation was supposed to be a major mechanism in WP1130-induced MM cell death, we next evaluated c-Maf ubiquitination and stability after WP1130 treatment. As shown in Figures 9c and d, WP1130 treatment resulted in c-Maf polyubiquitination and degradation. Because WP1130 also inhibits other deubiquitinases, such as USP9X,^14^ we wondered whether USP5 was important in WP1130-induced c-Maf degradation. To this end, RPMI-8226 cells were transfected with siUSP5 to knockdown USP5, followed by WP1130 treatment. As shown in Figures 9e and f, WP1130 markedly decreased c-Maf in the scramble group, in contrast, when USP5 was silenced, c-Maf degradation by WP1130 was not affected. These results suggested that WP1130-induced c-Maf degradation was USP5-dependent. Targeting at the USP5/c-Maf axis could be a potent strategy for MM treatment.

## Discussion

The above studies found that the deubiquitinase USP5 selectively interacts with and stabilizes c-Maf and MafB, two of the key members in the Maf transcription factor family, by suppressing their polyubiquitination. The functional studies revealed that USP5 is overexpressed in MM cells and upregulates c-Maf transcriptional activity, whereas inhibition of USP5 leads to MM cell apoptosis.

USP5 is a deubiquitinase that cleaves both linear and branched multi-ubiquitin polymers therefore has a key role in ubiquitin recycling and protein ubiquitination.^15^ It showed that inhibition of USP5 leads to accumulation of ubiquitin chains as that induced by proteasome inhibitors.^16^ However, its substrate proteins have not been largely identified and its biological functions are poorly understood. By searching public databases including the most popular PUBMED, one can find that there are two putative proteins, FOXM1 and the ion channel Cav3.2, which interact with and are stabilized by USP5.^17, 18^ The present study demonstrated that the transcription factors c-Maf and MafB are two new substrates that interact with USP5 and thus being stabilized by USP5 to prevent their ubiquitination. However, USP5 fails to interact with and stabilize MafA although these three members of the large Maf family share a high similarity in their amino-acid sequences and molecular structure. This finding further suggests that USP5 has a preference for its substrate proteins.

USP5 contains five specific domains, including the cryptic zinc finger (ZnF)-UBP, ZnF-UBP, the C-Box, the UBA1/UBA2, and the H Box domains. The ZnF domain interacts selectively with an unmodified C-terminus of the proximal ubiquitin and regulates deubiquitination,^12^ whereas the UBA domain is involved in polyubiquitin recognition and is proposed to limit ubiquitin chain elongation and to target polyubiquitinated proteins.^19^ It is reported that the ZnF-UBP domain binds to unanchored diglycine carboxyl tail,^15^ thus preventing substrate ubiquitination, whereas the cryptic ZnF-UBP domain is tightly bound to the catalytic core and is indispensable for the catalytic activity of USP5.^20^ In our present and previous studies, USP5 was found to reduce polyubiquitination modification of the Maf proteins.^11^ However, out of our expectation, the cryptic ZnF-UBP and the c-Box of USP5 can associate with Maf proteins as assayed by IP, which is different from previous findings on the association with ubiquitin moieties. This finding is also confirmed in the *in vitro* ubiquitination assay in which USP5 prefers to prevent c-Maf ubiquitination in the presence of an E3 ligase. Therefore, USP5 probably leads to deubiquitination by two means: (1) binding free ubiquitin chain and (2) removing bound ubiquitin chain from c-Maf. Moreover, the UBA1/UBA2 domain partly accumulates Maf proteins although this domain fails to interact with c-Maf, which could be explained by the possible quick interaction ('kiss and run') between c-Maf and the UBA1/UBA2 domain that could not be captured by our current methods.

Although it is less studied, USP5 has been suggested in regulating cell proliferation and apoptosis. The first target of USP5 is p53. As a main gatekeeper for cell survival/apoptosis, p53 is suppressed by USP5, whereas suppression of USP5 leads to p53 activation.^21^ In the present study, USP5 is found to be overexpressed in myeloma cells and stabilizes c-Maf and MafB, two key transcription factors in promoting MM cell proliferation and progression.^4^ USP5 expression leads to transactivation of the MARE-driving luciferase in the presence of c-Maf, which further results in the transcription of CCND2, ARK5, and ITGB7, three typical c-Maf downstream genes that promote MM cell proliferation and survival.^2, 4^ In contrast, USP5 knockdown leads to c-Maf degradation and MM cell apoptosis. Notably, suppression of USP5 by its inhibitor WP1130 leads to MM cell apoptosis in association with increased c-Maf ubiquitination and degradation. Although WP1130 probably inhibits several deubiquitinases including USP5, USP9X, and others,^14^ when USP5 was knocked down, the effects of WP1130 on MM cell death was markedly decreased, which suggested that USP5 is critical for WP1130 action in modulation of c-Maf stability and MM cell apoptosis. Moreover, both WP1130 treatment and USP5 knockdown prefer to induce apoptosis of MM cells that express endogenous c-Maf but display no apoptotic activity on c-Maf-negative MM cells. Therefore, there is a USP5/c-Maf axis in MM that promotes MM cell proliferation and survival. Inhibition of this axis leads to selective MM cell apoptosis.

In conclusion, the present study demonstrated that c-Maf and MafB proteins are substrates of USP5. By decreasing their ubiquitination and stabilizing their proteins, USP5 enhances their transcriptional activity and promotes MM cell proliferation. In contrast, inhibition of the USP5/c-Maf axis leads to MM cell apoptosis. Therefore, targeting the USP5/c-Maf axis is a novel potential strategy for the selective treatment of c-Maf-expressing MM.

## Materials and methods

### Cell culture

Human embryonic kidney cells (HEK293T) were maintained in Dulbecco’s modified Eagle’s medium (DMEM). MM cell lines including RPMI-8226, LP1, and U266 were obtained from Dr Aaron Schimmer, University of Toronto. MM cells were cultured in Iscove's Modified Dulbecco's Media. All media were supplemented with 10% of fetal bovine serum, glutamine and antibiotics.

### Plasmids

c-Maf was cloned from myeloma cell line LP1, the MafB and USP5 plasmids were purchased from Open Biosystems (Thermo Fisher, Waltham, MA, USA), whereas MafA was cloned from HeLa cells as described previously.^11^ To generate c-Maf mutants, all lysine (K) residues in c-Maf were mutated to Arginine (R), which generated K0.^8^ To obtain c-Maf mutants with single lysine residues, the corresponding R was recovered to K by site-directed mutagenesis as described previously.^8^ K308 c-Maf contained a single K residue, which was present at the 308th amino-acid site. All genes including ubiquitin were subcloned into a pcDNA3.1 vector carrying an HA, a Flag, or a Myc tag. Primers for the specific domains to generate USP5 truncates were designed as shown in Table 1. The Maf recognition element (MARE, 5′-TGCGAGTGAGGCA-3′) and its mutant version (mtMARE, 5′-**gta**GAGT**g**AG**tac**-3′) were obtained from a previous report.^4^ A nucleotide sequence containing six tandem MARE or mtMARE was chemically synthesized by Suzhou GeneWiz (Suzhou, China) and it was cloned into a pGL4 vector (Promega, Madison, WI, USA).

### Chemicals and antibodies

A polyclonal anti-c-Maf antibody was obtained from Santa Cruz Biotechnology, Inc., Santa Cruz, CA, USA. An anti-MafB antibody was purchased from Abgent (Suzhou, China). Monoclonal anti-HA, anti-Myc, anti-Flag, and anti-GAPDH antibodies were obtained from MBL (Tokyo, Japan). An anti-USP5 antibody was purchased from Proteintech (Chicago, IL, USA). MG132 was purchased from Santa Cruz Biotechnology, and cycloheximide (CHX) was purchased from Sigma-Aldrich Chemicals (St. Louis, MO, USA). WP1130 was purchased from Selleck Chemicals Inc. (Houston, TX, USA); HRP-labeled goat anti-mouse and goat anti-rabbit IgG (H+L) antibodies were purchased from Beyotime Institute of Biotechnology (Nantong, China). IRDye 680CW goat anti-mouse and IRDye 800CW goat anti-rabbit antibodies were from Odyssey (San Ramon, CA, USA).

### CHX chase assay

After transfected with plasmids of interest for 48 h, HEK293T cells were treated with CHX (100 *μ*g/ml) for 0 to 12 h. Cell lysates were then prepared by 2 × SDS lysis buffer, followed SDS-PAGE and IB analyses with specific antibodies as described previously.^8^

### Gene transfection

One day before transfection, HEK293T cells were seeded in six-well plates, when grown to 50% confluence, cells were subjected to gene delivery using polyethyleneimine (PEI) as described previously.^11^

### Immunoblotting

After transfection with appropriate plasmids, HEK293T cells were lysed on ice in a lysis buffer as described previously.^11^ After clarifying at high speed at 4°C, protein concentrations were determined by BCA assay (Pierce, Rockford, IL, USA). Equal amount proteins (30 *μ*g) were fractionated in SDS-PAGE, and transferred to polyvinylidene difluoride membrane. The blots were subjected to analysis against appropriate antibodies.

### Immunoprecipitation

HEK293T cells were transfected with USP5 and c-Maf, MafB or MafA plasmids for 48 h. All cells were treated with MG132 (40 *μ*M) for two hours before cell collection for IP assay as described previously.^11^

### In-tube ubiquitination assay

This protocol was adapted from a previous report.^22^ In brief, HA-c-Maf, HERC4, and Flag-USP5 plasmids were transfected into HEK293T cells, respectively. Forty-eight hours later, cells were treated with MG132 for 2 h, followed by cell lysate preparation. To enrich and purify c-Maf, HERC4, and USP5 proteins, individual cell lysates were subjected to IP with HA- (for c-Maf) or Flag- (for HERC4 and USP5) antibody-conjugating agarose beads, respectively, at 4 °C for 12 h. After that, the beads were washed four times with an IP lysis buffer, twice with 1 × ubiquitin reaction buffer (Boston Biochem, Boston, MA, USA) and then resuspended in 20 *μ*l of 1 × ubiquitin reaction buffer containing 200 ng of recombinant E1, 250 ng of recombinant UbcH5c, 10 *μ*g of ubiquitin, 0.5 mM ATP, and 1 × Energy Restoration System (Boston Biochem). The reaction was carried out at 30 °C for 2 h and then terminated by boiling in 2 × SDS loading buffer. Ubiquitinated products were resolved by SDS-PAGE and detected by IB analysis.

### Luciferase assay

The luciferase reporter plasmid pGL4-MARE.Luci or pGL4-mtMARE.Luci was co-transfected into HEK293T cells with USP5 and/or c-Maf. Forty-eight hours later, cell lysates were subjected to luciferase analysis using Bright-Glo system (Promega) as described previously.^11^ *β*-gal expression was applied as a transfection control. Luciferase activity was normalized to *β*-gal expression for each sample. All transfection experiments were performed in duplicates.

### Lentiviral USP5 construction

A human USP5 cDNA was inserted into the pLVX-AcGFP lentiviral vector (Clontech, Mountain View, CA, USA). To generate lentiviral particles, HEK293T cells at 80% confluence were transfected with 10 *μ*g of pLVX-AcGFP-USP5, 3.5 *μ*g of VSV-G envelope glycoprotein, 2.5 *μ*g of packaging proteins (Rev), and 6.5 *μ*g of packaging proteins (ΔR8.74) using PEI (Sigma) as a gene delivery carrier.^11^ After being washed and refreshed with the DMEM medium, cells were further cultured for 48 h. The lentiviral particle-enriched supernatant was harvested, filtered, and stored frozen at −80 °C. After titration, these lentiviral particles were applied to infect RPMI-8226 and LP1 cells for 96 h before being prepared for IP/IB assays.

### Knockdown with shRNA or siRNA of USP5

USP5 shRNAs (shUSP5, Genechem Co. Ltd., Shanghai, China) and HA-c-Maf were co-transfected into HEK293T cells and cultured for 48 h, cells were then prepared for IB assay. To knockdown USP5 or c-Maf in MM cell lines, USP5 or c-Maf siRNA double complexes (Ribobio Co. Ltd., Guangzhou, China) were transfected into MM cell lines by Lipofectamine 2000 (Invitrogen, Carlsbad, CA, USA). The most effective siRNA was chosen to transfect MM cells for 48 h, followed by IP/IB assay.

### Apoptosis detection with flow cytometric analysis

When cells were treated with WP1130 for 12 h or cells were knocked down of c-Maf or USP5 for 48–72 h, cells were collected for Annexin V-FITC/PI staining as the instructions from the manufacturer (MultiSciences Biotech Co., Ltd, Hangzhou, China) and subjected to analysis on a BD flow cytometer as described previously.^10^

### Cell proliferation assay by MTT assay

MM cells were infected with lentiviral shUSP5 for 1–7 days before being subjected to MTT (3-(4,5-dimethylthylthiazol-2-yl)-2,5-diphenyltetrazolium bromide) assay as described previously.^10^

### Reverse-transcription polymerase chain reaction (RT-PCR)

Total RNA was extracted using Trizol (Sangon Biotech, Shanghai, China). RNA (2 *μ*g) was reverse-transcribed using an EasyScipt First-strand cDNA Synthesis (Vanzyme, Nanjing, China) according to the manufacturer’s instruction. PCR amplification was carried out using the following primers: for USP5, 5′-CGGATTTGACCTTAGCG-3′ (Forward) and 5′-CTGCCATCGAAGTAGCG-3′ (Reverse); for GAPDH, 5′-AATCCCATCACCATCTTCC-3′ (Forward) and 5′-CATCACGCCACAGTTTCC-3′ (Reverse); for CCND2, 5′-ATTTACACCGACAACTCCATC-3′ (Forward) and 5′-CTCAGTCAGGGCATCACAA-3′ (Reverse); for ITGB7, 5′-GACGCCAAGATCCCATCC-3′ (Forward) and 5′-GGGTATCCCTCAGCACGAA-3′ (Reverse); for ARK5, 5′-GTCCTGCCTTACCCTCTACT-3′ (Forward) and 5′-CAGGCTCTGACAGGGATT-3′ (Reverse). The PCR products were visualized by Goldview staining (Transgen, Beijing, China), following electrophoresis on 2% agarose gels.

### GEO data set analyses

The DNA microarray data set from patients with primary plasma cell malignancies was retrieved from Oncomine databases (https://www.oncomine.org/) and the data set was reported from Chapman MA *et al.*^23^ Log_2_(USP5 mRNA level) was reported. Statistical difference between the control and each patient group was analyzed by student’s *t-*test.

### Statistics

Statistical difference between the control and the experimental groups was analyzed by student’s *t-*test.

## Publisher’s Note:

Springer Nature remains neutral with regard to jurisdictional claims in published maps and institutional affiliations.

## Figures and Tables

**Table 1:** Primers for constructs of USP5 domains

**Domain**	**a.a. region**	**Size (bp)**	**Primers (5'-3')**
D1 Cryptic ZnF	1–168	504	F: ATTGGATCCATGGCGGAGCTGAGTGAGGAGGCGCTGCTR: CCGCTCGAGTTATGCCTGCACCTCCTGCTTGCG
D2 ZnF	169–289	363	F: ATTGGATCCTGGGATGGGGAAGTACGGCAGR: CCGCTCGAGTTATGTCTTCTGCATCTTCAGCATGTC
D3 C-Box	290–624	1005	F: ATTGGATCCGACAAGACGATGACTGAGTTGR: CCGCTCGAGTTACGGAGTGACCAGGGGT
D4 UBA1–UBA2	625–749	375	F: ATTGGATCCGATGAGCCCAAAGCGCCCATR: CCGCTCGAGTTAGGAGCCCGGCCCACTAGA
D5 H Box	750–835	258	F: ATTGGATCCACAAGCGCAGCAGCCGACR: CCGCTCGAGTTAGCTGGCCACTCTCTGGTAGAAGTAGAT

Underlined sequences are restriction enzyme recognition sites: GGATCC for *Xho*I, CTCGAG for *BamH* I.
